# Advances in research on biomarkers associated with acute myocardial infarction: A review

**DOI:** 10.1097/MD.0000000000037793

**Published:** 2024-04-12

**Authors:** Xuelan Huang, Suwen Bai, Yumei Luo

**Affiliations:** a Guangdong Medical University, Zhanjiang, China; b Central Laboratory, The Second Affiliated Hospital, School of Medicine, The Chinese University of Hong Kong, Shenzhen & Longgang District People’s Hospital of Shenzhen, Shenzhen, China; c Cardiology Department of The Second Affiliated Hospital, School of Medicine, The Chinese University of Hong Kong, Shenzhen & Longgang District People’s Hospital of Shenzhen, Shenzhen, China.

**Keywords:** acute myocardial infarction, biomarkers, H-FABP, microRNA, troponin

## Abstract

Acute myocardial infarction (AMI), the most severe cardiovascular event in clinical settings, imposes a significant burden with its annual increase in morbidity and mortality rates. However, it is noteworthy that mortality due to AMI in developed countries has experienced a decline, largely attributable to the advancements in medical interventions such as percutaneous coronary intervention. This trend highlights the importance of accurate diagnosis and effective treatment to preserve the myocardium at risk and improve patient outcomes. Conventional biomarkers such as myoglobin, creatine kinase isoenzymes, and troponin have been instrumental in the diagnosis of AMI. However, recent years have witnessed the emergence of new biomarkers demonstrating the potential to further enhance the accuracy of AMI diagnosis. This literature review focuses on the recent advancements in biomarker research in the context of AMI diagnosis.

## 1. Introduction

Cardiovascular diseases (CVDs) have become the “first killers” worldwide and pose substantial threats to public health. The Report on Cardiovascular Health and Diseases in China 2022 points out that the prevalence of CVD and mortality rate in China is still in a continuous rising stage, projecting that the number of people suffering from CVD currently reaches 330 million cases, and two out of 5 deaths are due to CVD.^[1]^ Acute myocardial infarction (AMI) is the primary cause of mortality among various CVDs, and the incidence and death rates have been steadily increasing. AMI is a life-threatening condition characterized by rapid progression and a narrow window for optimal treatment, leading to low rescue efficiency and consequent elevations in disability and mortality rates. With the increasing sophistication of thrombolysis, reperfusion, and interventional techniques, the mortality rate of AMI has declined in developed countries, but it remains one of the leading causes of morbidity and mortality worldwide.^[2]^ Therefore, the exploration and development of novel rapid and early diagnostic methods hold substantial promise for enhancing patient survival and prognosis. Currently, the diagnosis of AMI is primarily based on electrocardiogram (ECG) specific changes, typical chest pain, and abnormal myocardial markers. The ECG is the first step in recognizing STEMI (ST-segment elevation myocardial infarction), which will help in deciding percutaneous coronary intervention or thrombolysis, and in these patients biomarker testing is usually not needed. Biomarkers are useful for atypical clinical presentation and nonspecific ECG abnormalities. Creatine kinase isoenzymes (CK-MB), myoglobin (Mb) and troponin are routinely used in the management of STEMI and non-ST elevation myocardial infarction (NSTEMI).^[3]^ In patients with atypical clinical symptoms and NSTEMI, the diagnosis must be confirmed by relying on changes in biomarkers of myocardial injury. Blood biomarker measurement serves as a crucial diagnostic tool for AMI, it is important in the diagnosis of myocardial infarction and can help to make an earlier diagnosis and start specific treatment. Notably, cardiac troponin I (cTnI) and cardiac troponin T (cTnT) are extensively recognized as the most critical indicators of myocardial injury due to their high sensitivity and specificity, making troponins the best biomarkers for diagnosis and prognosis.^[4]^ Recent years have witnessed impressive growth in our understanding of AMI-associated biomarkers detectable in tissue, peripheral blood, urine, and saliva. The emergence of novel and promising biomarkers has opened new avenues in the predictive diagnostics of AMI, such as miRNAs (miRNAs, miRs), H-FABP (heart-type fatty acid-binding protein), urinary cTns, salivary cTns, and other biomarkers used in the early diagnosis of AMI. This review presents an overview of recent advancements in AMI biomarker research and highlights the potential of these diagnostic tools.

## 2. MI overview

AMI arises from the obstruction of coronary arteries, resulting in insufficient blood supply and myocardial necrosis in the affected area. According to the 2018 revision of the Fourth Universal Definition of Myocardial Infarction,^[5]^ the diagnostic criteria for MI depend on the detection of acutely injured myocardium with clinical symptoms of AMI and a detectable rise or fall in cardiac troponin levels, with at least one value exceeding the 99th percentile upper reference limit. Furthermore, confirmation of AMI requires the fulfillment of at least one of the following conditions: symptoms of myocardial ischemia, new ischemic changes on ECG, appearance of pathological Q-waves, imaging evidence of recent loss in myocardial activity or new regional ventricular wall motion abnormalities, or identification of intracoronary thrombus using coronary angiography or autopsy examination. While ECG changes, typical chest pain, and abnormal myocardial markers are primary diagnostic tools for AMI, the identification of biochemical markers assumes a critical role in diagnosing patients with AMI who manifest atypical symptoms and inconspicuous ECG changes, thereby facilitating a more accurate assessment of myocardial damage.^[6]^

## 3. Conventional biomarkers for AMI

The pioneering work of Ladue et al in 1955 marked a significant milestone by identifying aspartate aminotransferase (AST) levels as potential indicators for diagnosing AMI. This remarkable discovery established AST as the first reported biochemical marker for AMI diagnosis and laid the foundation for further research on myocardial injury-related markers.^[7]^ Subsequently, alterations in blood lactate dehydrogenase (LDH) and creatine kinase (CK) levels emerged as additional indicators of AMI.^[8]^ In 1979, the World Health Organization recommended myocardial enzyme tests, including AST, LDH, and CK, as biological markers for the diagnosis of AMI. Nevertheless, these myocardial enzyme profiles mainly reflect enzyme activities, which are not exclusively confined to the heart and can also be detected in other organs, such as skeletal muscle and liver. Therefore, these myocardial enzyme profiles have been discontinued from AMI diagnostic criteria due to their lack of heart specificity.

In contrast, CK-MB, an isozyme of CK, has demonstrated superior diagnostic accuracy compared to AST, LDH, and CK, rendering it one of the most widely used serum enzyme markers in clinical practice. Despite its diagnostic benefits, CK-MB lacks specificity.^[9]^ The diagnostic approach to AMI has undergone significant transformation over the years, driven by ongoing research into myocardial markers and significant advancements in detection techniques. Initially, the identification of MI relied on assays measuring enzyme activities, including cTns, AST, LDH, and CK-MB. However, the field has shifted towards assays quantifying protein mass concentrations, such as Mb and CK-MB mass. In 2000, the World Health Organization recommended cTn as the primary biomarker for diagnosing AMI.^[10]^ In clinical practice, the most commonly used biomarkers for AMI diagnosis include cTns, Mb, and CK-MB. Notably, high-sensitivity cardiactroponin assays have revolutionized the diagnostic process by offering enhanced sensitivity and shortened diagnostic time. Accordingly, high-sensitivity cardiactroponin (hs-cTn) assays have been widely applied in the early detection and management of AMI in clinical settings.

### 3.1. Troponins for AMI diagnosis

**Figure 1.**
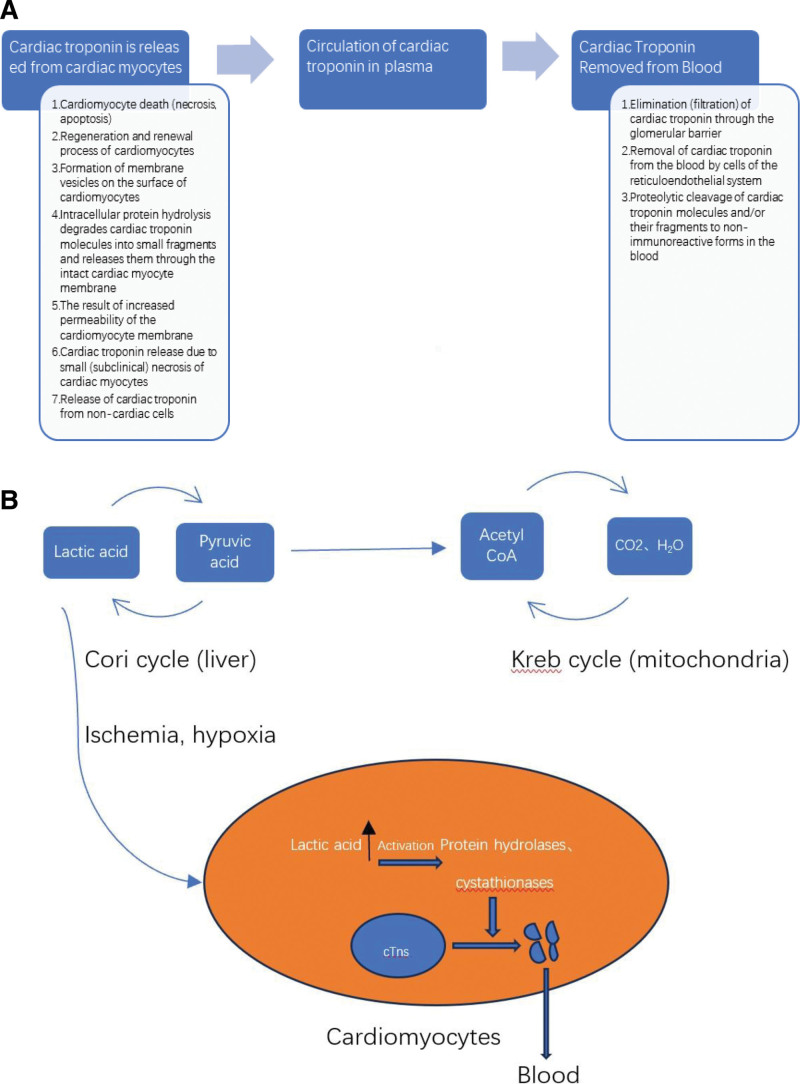
Metabolic pathway of cTns. The metabolic pathway of cTns consists of 3 main stages: release of cTn from cardiomyocytes, circulation of cTn in plasma, and removal of cTn from circulation. cTn = cardiac troponin.

Interestingly, cTns are important proteins in cardiomyocytes that play a critical role in regulating myocardial contraction and relaxation. These proteins comprise 3 distinct subunits: troponin C (TnC), TnI, and TnT.^[11]^ Each subunit has specific regulatory functions in cardiac muscle contraction and diastole. TnC is a calcium-binding subunit that induces complex conformational changes. TnT is a tropomyosin-binding subunit that binds the entire troponin molecule to tropomyosin. Conversely, TnI is an inhibitory subunit, mainly responsible for repressing actin-myosin interactions.^[12]^ The cTn metabolic pathway involves 3 key stages: release of cTn from cardiomyocytes, circulation of cTn in plasma, and removal of cTn from circulation^[13,14]^ (Fig. 1). Notably, cTn is retained in intact membranes of cardiomyocytes and cannot be released into the bloodstream. Hence, plasma concentrations of cTnT and cTnI remain undetectable or markedly low in individuals without myocardial damage. Upon cardiomyocyte injury, cTnI and cTnT become detectable in the peripheral bloodstream. Typically, their concentrations start to rise within 3 to 4 hours after myocardial injury and reach a peak at 12 to 24 hours post-injury. The elevated levels of cTnI and cTnT are sustained for 7 to 10 days, with cTnT levels remaining elevated for 10 to 14 days. Nevertheless, the challenge in utilizing these biomarkers for diagnostic purposes arises from the structural similarity of TnC between cardiac and skeletal muscles, which complicates the differentiation between myocardial and skeletal muscle damage based solely on blood levels of TnC. Therefore, the use of blood cTnC level as a diagnostic biomarker for AMI is complicated.^[15]^ Unlike cTnC, the amino acid composition of cTnI and cTnT proteins exhibit marked differences, with over 40% difference in their amino acid sequences when compared to their counterparts in skeletal muscle.^[16]^ This difference underscores the specificity of cTnT and cTnI as biomarkers in the clinical laboratory diagnosis of myocardial injury.^[17]^ Particularly, cTnI is highly specific to the myocardium and is minimally present in skeletal muscle, which elevates its diagnostic sensitivity and specificity beyond those of conventional myocardial enzyme profiles. Consequently, cTnI has emerged as the definitive “gold standard” and preferred biomarker for diagnosing AMI.^[18]^ Beamish et al^[19]^ have demonstrated the superiority of cTnI as a biomarker in terms of sensitivity and specificity, leading to its widespread adoption for diagnosing NSTEMI. Despite its diagnostic superiority, the utility of cTnI is constrained by the delayed increase in its levels in peripheral blood. Hence, repeated blood tests are necessary to exclude AMI, rendering cTnI less effective for diagnostic purposes beyond the initial 2 weeks following an AMI event. Therefore, cTnI is not routinely used for early diagnosis of AMI. Instead, it is often used in conjunction with Mb in clinical settings to enhance diagnostic accuracy during the early stages of AMI detection. The recent introduction of the high-sensitivity cTn assay enables the detection of extremely low levels of high-sensitivity cTnI, down to a few ng/L in human body fluids.^[20]^ This advancement leads to a significant reduction in the diagnostic time for AMI and provides higher accuracy than conventional cTn assays. Thus, the high-sensitivity cTnI assay is widely used in clinical practice due to its exceptional precision and the ability to provide rapid diagnosis of AMI. Elevated cTn levels are observed in various cardiac and non-cardiac conditions beyond the diagnosis of AMI. These conditions include heart failure (HF), various forms of myocarditis, outcomes of catheter ablation, coronary revascularization procedures, pulmonary hypertension, stroke, subarachnoid hemorrhage, pulmonary embolism, subarachnoid hemorrhage, conditions affecting critically ill patients, chronic diseases, adverse reactions to chemotherapy, and physiological responses to strenuous exercise^[21–42]^ (Table 1). Therefore, novel early biomarkers with high sensitivity and specificity are urgently required to further reduce the mortality associated with AMI.

**Table 1:** Possible causes of elevated serum cTn concentrations.

Cardiac disease	1. Heart failure	^[21]^
	2. Inflammatory heart disease (endocarditis, myocarditis, pericarditis)	^[22]^
	3. Cardiomyopathy (all types such as hypertrophic cardiomyopathy)	^[23]^
	4. Aortic coarctation	^[24]^
	5. Arrhythmias (atrial fibrillation, supraventricular tachycardia, etc.)	^[25,26]^
	6. Cardiotoxic drugs (most chemotherapy drugs such as anthracyclines, alkylating agents, cocaine, etc.)	^[27]^
	7. Heart contusion	^[28]^
	8. Cardiac surgery (coronary revascularization, procedures other than revascularization, defibrillation discharge, catheter ablation, etc.)	^[32,34]^
	9. Takotsubo syndrome	^[5]^
Myocardial injury in non-cardiac and systemic pathologies	1. Strenuous exercise	^[29]^
	2. Stress and Stimulation	^[33]^
	3. Chronic diseases (chronic kidney failure, COPD, diabetes, high blood pressure, oncology.)	^[30,31,36,37]^
	4. Pulmonary embolism	^[35]^
	5. Diseases of the central nervous system (e.g., stroke, subarachnoid hemorrhage)	^[42]^
	6. Critically ill patients (severe anemia, systemic inflammatory response, respiratory diseases, etc.)	^[41]^
	7. Covid-19	^[40]^
False positive causes of elevated troponin	1. Analyzer failure 2. Interference from fibrin or endogenous antibodies such as rheumatoid factor or heterophilic antibodies.	^[38,39]^

cTn = cardiac troponin.

### 3.2. Mb for AMI diagnosis

Mb is an oxygen-bound heme protein consisting of a polypeptide chain and a heme cofactor. It contains 153 amino acids and has a molecular weight of 16.8 kDa. Mb serves as an intramuscular oxygen storage protein that belongs to the group of globin proteins found in cardiac and skeletal muscles. It derives its name from its structural and functional similarity to hemoglobin.^[43]^ Mb is an iron carrier involved in the transport and binding of oxygen and is stored in the cytoplasm of skeletal and cardiac muscle cells. Upon cardiomyocyte damage in the event of an AMI, Mb is rapidly released into the bloodstream. Due to its small molecular size, renal clearance of Mb occurs within 24 hours.^[44]^ Mb is typically found at very low concentrations in normal serum. However, its levels began to rise 1 hour after AMI onset, peaking within 12 hours. Subsequently, they returned to baseline within 1 to 2 days^[45]^ (Table 2). Moreover, Mb is regarded as the most sensitive indicator for early prediction of AMI.^[46]^ Nevertheless, its specificity is limited owing to the influence of skeletal muscle disease and renal dysfunction on its concentration.^[47]^ Hence, the use of Mb alone in diagnosing AMI is insufficient, and it is typically combined with other indicators, such as cTns, CK-MB, ECG, and clinical symptoms. Because of its high sensitivity, Mb is typically used to rule out AMI.^[48]^ Furthermore, Mb is also used to diagnose re-infarction in patients with AMI and to determine the extent of the infarct, serving as a biomarker for assessing ischemia-reperfusion injury.^[49]^

**Table 2:** Dynamic changes in markers of myocardial injury.

Myocardial injury markers	Start rise time (h)	Peak time (h)	Duration (d)	Normal value	Threshold value
Mb	1	12	1–2	50–85 μg/L	>75 μg/L
CK-MB	4	16–24	3–4	10–25 U/L	>25 U/L
cTnI	3–4	11–12	7–10	˂0.2 μg/L	>1.5 μg/L
cTnT	3–4	24–48	10–14	0.02–0.13 μg/L	>0.2 μg/L

CK-MB = creatine kinase isozyme – MB, cTnI = cardiac troponin I, cTnT = cardiac troponin T, Mb = myoglobin.

### 3.3. CK isoenzyme for AMI diagnosis

**Figure 2.**
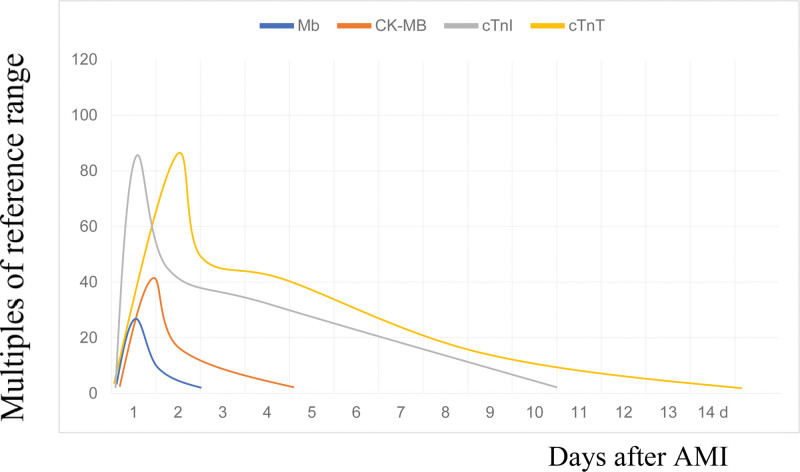
Time-dependent changes in serum concentrations of different myocardial injury markers after an AMI. The temporal characteristics of troponin, Mb, and CK-MB in blood after AMI are illustrated. AMI = acute myocardial infarction, CK-MB = creatine kinase isozyme-MB, cTnI = cardiac troponin I, cTnT = cardiac troponin T, Mb = myoglobin.

CK, previously referred to as creatine phosphokinase, is a membrane transport protein involved in cytoplasmic energy metabolism. It is a dimeric enzyme comprising muscle and brain subunits and catalyzes the production of adenosine triphosphate.^[50]^ The distribution of CK is widespread across various tissues but is particularly concentrated in the skeletal muscle, heart muscle, and brain tissues. In these tissues, 3 different isozymes of CK are found: CK-MB, CK-BB, and CK-MM.^[51]^ Of these, CK-MB is widely distributed in cardiomyocytes and accounts for 14% to 42% of the total CK in the myocardium. It is a specific biomarker of heart injury and is considered the “gold standard” for diagnosing AMI.^[52]^ After myocardial injury, CK-MB is rapidly released from myocardial tissue into the serum, with levels increasing within 4 hours of AMI onset. The peak concentration occurred at 16 to 24 hours and returned to normal within 3 to 4 days (Fig. 2). Despite its cardiac association, CK-MB is not specific to the heart. It may also be released due to injuries in skeletal muscles, liver, stomach, and uterus, resulting in false-positive diagnoses when these tissues are damaged.^[53]^ Consequently, CK-MB is rarely used alone for the diagnosis of AMI, especially in the clinical application of cTn and other biomarkers. However, it retains value in detecting AMI and can be employed for post-AMI condition monitoring and prognostication. Furthermore, the proportionality of serum CK-MB levels to the severity of myocardial damage provides a quantitative measure of the extent of cardiac injury.^[54]^ Finally, CK-MB can also serve as an indicator of ischemia-reperfusion injury.^[55]^

## 4. Emerging biomarkers associated with AMI

The identification of an ideal biomarker of AMI includes several critical characteristics. First, the biomarker should be accessible through minimally invasive methods, such as plasma, serum, urine, or saliva samples. Second, it must demonstrate high sensitivity and specificity, manifest early in the diagnostic process, and remain detectable over an extended period. Third, the biomarker should facilitate rapid, accurate, and cost-efficient testing. Despite extensive research in the field of AMI diagnostics, a biomarker that meets all these stringent criteria remains elusive. This gap underscores the imperative need for the identification, testing, and clinical utilization of novel markers. Recent advancements have led to the identification of several promising biochemical markers of AMI. These markers include blood microRNAs (miRNAs, miRs), H-FABP, high-sensitivity C-reactive protein (hs-CRP), B-type natriuretic peptide, urinary cTn, and salivary cTn.

### 4.1. miRNAs associated with AMI

MiRNAs are a class of non-coding single-stranded small RNA molecules with a length of approximately 20 to 24 nucleotides. These endogenous, nonprotein-coding molecules have garnered attention in recent scientific research due to their evolutionary conservation and pivotal regulatory functions.^[56]^ MiRNAs play important regulatory roles in biological evolution and disease development. They confer effects by specifically binding to the 3′-end untranslated regions of target messenger RNAs and affect their degradation and translational repression at the post-transcriptional level to regulate gene expression.^[57]^ In vitro, miRNAs contribute significantly to cellular processes, such as proliferation, differentiation, apoptosis, and other physiopathological processes.^[58,59]^ Furthermore, miRNAs are involved in the development of CVDs, including coronary artery disease. In 2009, researchers identified stable levels of circulating miRNAs, since then, the potential of circulating miRNAs as disease biomarkers has been recognized.^[60]^ Subsequent research has illuminated the predictive value of miRNAs for various diseases, including AMI, acute coronary syndrome (ACS), HF, hypertensive disorders, and stroke.^[61]^

The potential of miRNAs to serve as sensitive biomarkers for early diagnosis of AMI can be attributed to their release from the injured myocardium into the bloodstream during AMI. These circulating miRNAs can be easily detected and exhibit remarkable stability in various bodily fluids, including whole blood, plasma, serum, and urine.^[62,63]^ MiRNAs are characterized by a simple structure, low complexity, and tissue-specific expression. Unlike conventional protein markers, miRNAs do not undergo post-transcriptional modifications, rendering them highly detectable. Furthermore, miRNAs are easily detectable after AMI with greater accuracy than conventional protein markers. Key heart-specific miRNAs, such as miR-208a/b, miR-499, miR-1, and miR-133a, are highly expressed in cardiac muscles and play important roles in cardiac physiology.^[64–66]^

Interestingly, miR-208a exhibits robust cardiac specificity and remains undetectable in healthy individuals and patients without AMI. However, miR-208 is upregulated in the first hour following AMI, with a 100% detection rate in plasma within 4 hours, surpassing the detection rate of cTnI (85%).^[67]^ Therefore, miR-208a is expected to be a sensitive marker of AMI with superior sensitivity compared to troponin. A study by Chen et al found high expression of miR-208a in 84 patients with AMI compared to 50 healthy controls. This finding suggests the potential of miR-208a as a promising biomarker for the diagnosis and prediction of AMI, thus positioning it as a highly valuable biomarker in clinical settings.^[68]^ Multiple studies have shown that circulating miR-208 is a reliable biomarker for diagnosing STEMI and NSTEMI. Moreover, miR-208 can distinguish patients presenting with AMI symptoms, such as chest pain, from healthy controls.^[69]^ A cohort study involving 1000 patients also confirmed an increase in circulating levels of miR-208b, miR-499, and miR-320a in patients with AMI. Among these miRNAs, miR-208b exhibited the highest diagnostic accuracy for AMI, as evidenced by its area under the receiver operating characteristic curve of 0.76 (95% confidence interval: 0.72–0.80). While the diagnostic efficiency of miR-208b was slightly lower than that of cTnI, it still emerged as a valuable diagnostic indicator.^[70]^

Furthermore, a rat model of AMI showed significantly elevated plasma miR-499 levels.^[71]^ According to a meta-analysis, circulating miR-499 has considerable diagnostic value in the detection of AMI.^[72]^ Notably, miR-499 displays enhanced accuracy, especially in the diagnosis of acute NSTEMI in the older population, surpassing conventional cTnT.^[73]^ The expression levels of miR-208b and miR-499 were significantly higher in patients with AMI than in healthy controls. Although their diagnostic specificity and sensitivity for AMI are comparable to those of cTnT, they do not outperform the diagnostic performance of cTnT.^[74]^ In a meta-analysis of Liu et al, 9 studies on miR-1 and 8 studies on miR-499 and miR-208 were analyzed. Although miR-1, miR-208, and miR499 all showed high predictive value, the ROC analysis revealed that cardiac-specific miR-208 and miR-499 served as more reliable biomarkers for diagnosis and prediction of AMI than miR-1.^[75]^ Ai et al^[76]^ reported significantly higher circulating miR-1 levels in patients with STEMI than in healthy controls. These findings were corroborated by Su et al,^[77]^ who observed markedly higher circulating miR-1 levels in the AMI group compared to the non-AMI group, indicating its potential diagnostic value for AMI. In an animal model of STEMI, circulating miR-133a levels were found to increase rapidly 1 hour after the onset of symptoms, peaking at 3 hours thereafter, similar to the pattern observed for TnI. Although the levels of miR-133a displayed a gradual rise, they consistently remained lower than those of TnI.^[78]^ Additionally, serum miR-208 and miR-499 expression levels were notably elevated in the AMI group relative to the control group at various time points.

However, these miRNAs did not demonstrate superior diagnostic performance compared to hs-TnI in the early diagnosis of AMI.^[79]^ Li et al^[80]^ also presented findings indicating that miR-1, miR-133a, miR-208b, and miR-499 may be promising biomarkers for the diagnosis of AMI; however, they do not exhibit superior diagnostic capabilities compared with cTnT. Several studies have uncovered the presence of miRNAs in the urine of patients with AMI, suggesting their potential as novel biomarkers for AMI.^[81,82]^ Furthermore, several other miRNAs, such as miR-203, miR-19a, miR-181a, miR-142, miR-22-5p, miR-122-5p, miRNA-124, and miR-363-3p, have been identified to be significantly differentially expressed in patients with AMI.^[83–122]^ A summary of various miRNAs implicated in the diagnosis of AMI is shown in Table 3. However, there is limited evidence to validate their diagnostic potential, and additional studies are required for further confirmation.

**Table 3:** Potential miRNA biomarkers for the diagnosis of AMI.

miRNA	Species	Source	Expression	Detection method	Year	References
miR-208	Rat	Urine	UP	qRT-PCR	2012	^[81]^
miR-208	Human	Plasma	UP	qRT-PCR	2015	^[75]^
miR-208a	Human	Serum	UP	qRT-PCR	2022	^[68]^
miR-208b	Human	peripheral blood	UP	qRT-PCR	2020	^[74]^
miR-499	Human	Plasma	UP	qRT-PCR	2020	^[83]^
miR-499a	Human	Serum	UP	qRT-PCR	2018	^[84]^
miR-499a-5p	Human	Plasma	UP	qRT-PCR	2021	^[85]^
miR-499-5p	Human	Plasma	UP	TaqMan®MicroRNA	2018	^[86]^
miR-1	Human	peripheral blood	UP	qRT-PCR	2019	^[77]^
miR-133	Human	Plasma	UP	qRT-PCR	2014	^[87]^
miR-133a miR-133b	Human	Serum	UP	qRT-PCR	2015	^[88]^
miR-363-3p	Rat	Serum	UP	qRT-PCR	2020	^[89]^
miR-124	Human	Serum	UP	qRT-PCR	2017	^[90]^
miR-122-5p	Human	Plasma	UP	qRT-PCR	2018	^[93]^
miR-22-5p	Human	Plasma	Down	qRT-PCR	2018	^[93]^
miR-19a	Human	Serum	UP	qRT-PCR	2020	^[92]^
miR-223 miR-483-5p	Human	peripheral blood	UP	qRT-PCR	2019	^[91]^
miR-1-3p miR-19b-3p miR-223-3p	Human	Plasma	UP	qRT-PCR	2021	^[85]^
miR-134	Human	Plasma	UP	qRT-PCR	2014	^[94]^
miR-328	Human	Plasma	UP	qRT-PCR	2020	^[71]^
miR126-3p	Human	Plasma	UP	qRT-PCR	2017	^[95]^
miR-134-5p miR-186-5p	Human	Plasma	UP	qRT-PCR	2016	^[96]^
miR-183-5p	Human	Plasma	UP	qRT-PCR	2018	^[101]^
miR-134-5p miR-15a-5p let-7i-5p	Human	Plasma	Down	qRT-PCR	2018	^[101]^
miR-26a miR-191	Human	Plasma	Down	qRT-PCR	2015	^[98]^
miR-150 miR-150-3p miR-486	Human	Plasma	UP	qRT-PCR	2015	^[104]^
miR-486-3p	Human	Serum	UP	qRT-PCR	2014	^[97]^
miR-126	Human	Plasma	Down	qRT-PCR	2012	^[100]^
miR-126-3p miR-26a-5p miR-191-5p	Human	Serum	Down	qRT-PCR	2014	^[97]^
miR-26a-1 miR-146a miR-199a-1	Human	Plasma	UP	qRT-PCR	2019	^[103]^
miR-181a miR-181c	Human	Plasma	UP	qRT-PCR	2016	^[112]^
miRNA-203	Human	Serum	UP	qRT-PCR	2022	^[99]^
miR-122 miR-375	Rat	Plasma	Down	qRT-PCR	2010	^[78]^
miR-451a	Human	peripheral blood	UP	qRT-PCR	2015	^[70]^
miR-486-5p miR-21-5p	Human	peripheral blood	Down	qRT-PCR	2015	^[70]^
miR-361-5p miR-21-5p	Human	Plasma	UP	qRT-PCR	2014	^[102]^
miR-519e-5p	Human	Plasma	Down	qRT-PCR	2014	^[102]^
miR-221-3p	Human	Plasma	UP	qRT-PCR	2016	^[106]^
miR-30a miR-30d miR-423-5p	Human	peripheral blood	UP	qRT-PCR	2015	^[107]^
miR-195	Human	Plasma	UP	qRT-PCR	2012	^[109]^
miR-497	Human	Plasma	UP	qRT-PCR	2014	^[108]^
miR-22	Human	Plasma	UP	qRT-PCR	2020	^[83]^
miR-25-3p miR-374b-5p miR-25-3p	Human	Plasma, Platelets	UP	qRT-PCR	2013	^[111]^
miR-92a-3p miR-30d-5p miR-186-5p miR-342-3p miR-374b-5p	Human	Plasma, Platelets	Down	qRT-PCR	2013	^[111]^
miR-139-5p	Human	Serum	UP	qRT-PCR	2021	^[110]^
miR-6718 miR-4329	Human	Serum	Down	qRT-PCR	2021	^[105]^
miR-331 miR-151-3p	Human	Plasma	UP	qRT-PCR	2020	^[117]^
miR-32-5p	Human	Serum	UP	qRT-PCR	2020	^[116]^
miR-379	Human	Plasma	Down	qRT-PCR	2018	^[120]^
miR-206	Rat, Human	Cardiac tissue	UP	qRT-PCR	2009	^[118]^
miR-21 miR-214 miR-233	Human, Rat	Cardiac tissue	UP	qRT-PCR	2008	^[119]^
miR-29b miR-149	Human, Rat	Cardiac tissue	Down	qRT-PCR	2008	^[119]^
miR-21	Human	Plasma	UP	qRT-PCR	2016	^[121]^
miR-1909-5p	Human	Plasma	UP	qRT-PCR	2016	^[115]^
miR-941	Human	Plasma	UP	qRT-PCR	2017	^[113]^
miR-145	Human	Plasma	Down	qRT-PCR	2017	^[122]^
miR-142	Human	Serum	UP	qRT-PCR	2020	^[114]^

AMI = acute myocardial infarction.

### 4.2. H-FABP associated with AMI

H-FABP, a small cytosolic protein (15 kDa), is involved in fatty acid transport in cardiac muscle cells. This protein is predominantly present in high concentrations in the cytoplasm of cardiomyocytes, with lower levels found in other tissues and organs, such as skeletal muscle and kidneys. Due to its low molecular weight, high concentration in cardiomyocytes, and water solubility, H-FABP is easily and promptly released from the cytoplasm into the bloodstream following cardiac injury. Furthermore, it is primarily eliminated from the plasma through renal excretion.^[123]^ Notably, the concentration of H-FABP increases rapidly (<1 hour) during myocardial injury and can be detected clinically, peaking at 4 to 6 hours thereafter and returning to baseline within 24 hours.^[124]^ These characteristics suggest that H-FABP may serve as a rapid diagnostic biomarker for the early detection of AMI.^[125]^ Several national and international clinical studies have consistently demonstrated the superior accuracy and sensitivity of H-FABP over other myocardial biomarkers, including TnI and Mb, in the prediction of AMI. Specifically, H-FABP has shown superior potential for diagnosing AMI, particularly in the early stages of AMI. In a study of 89 patients with suspected ACS, laboratory and point-of-care testing were performed to detect H-FABP, TnI, and CK-MB levels. The results underscored that among patients who presented within 4 hours after the onset of symptoms, H-FABP had a significantly higher diagnostic accuracy for AMI than the other cardiac parameters. Hence, the identification of H-FABP is important for the early diagnosis of AMI and the prediction of myocardial injury.^[126]^ McCann et al analyzed 664 patients admitted with acute ischemic chest pain. Their observations revealed that H-FABP demonstrated significantly greater sensitivity than cTnT for detecting AMI in patients within 4 hours of symptom onset.^[127]^ The study included 138 patients with suspected AMI, 95 patients with confirmed AMI, and 43 patients without AMI. Serum CK-MB, cTnT, and H-FABP levels were measured at different time points after AMI (<3, 3–6, and 6–12 hours). The results highlighted a notable increase in serum H-FABP levels in the AMI group at <3 hours post-AMI, with further increases observed at 3–6 and 6–12 hours after AMI. This finding indicates that the diagnostic value of the myocardial injury marker H-FABP was superior to that of conventional myocardial markers for early AMI (<3 and 3–6 hours post-AMI).^[128]^ In a case-control study using enzyme-linked immunosorbent assay, H-FABP levels were significantly correlated with selected markers, including CK-MB, hs-CRP, and TnT. The study involved the measurement of serum H-FABP, CK-MB, and cTnT levels and an immunoturbidimetric assay to determine hs-CRP levels in patients with or without AMI.^[129]^ Compared with cTn, H-FABP is emerging as a diagnostic marker for the early acute phase, demonstrating its utility in excluding patients without AMI. Therefore, it serves as a superior tool for diagnosing AMI, particularly during the early stages.^[130]^ In particular, the combined detection of H-FABP, hs-cTnI, and MB has shown the potential to compensate for the limitations of individual tests and prominently improve the diagnostic accuracy of AMI. Moreover, H-FABP has also been identified as an important factor in both the treatment and prognosis of AMI.^[131]^

### 4.3. Urine cTns associated with AMI

Urine, the final metabolic product of blood, is formed through a complex process involving glomerular filtration, tubular and collecting ductal resorption, elimination, and excretion. This intricate process yields a large amount of biological information, with changes in its composition, volume, and properties serving as reflective indicators of the body’s overall metabolic status. Notably, urine offers distinct advantages compared with other body fluid samples such as serum. Urine collection is noninvasive, convenient, and easily preservable. Furthermore, its protein composition is relatively simple and easy to analyze, rendering it a promising source of biomarkers.^[132,133]^ Despite these advantages, the collection of urine samples in an acute setting poses a challenge in contrast to blood sample acquisition. At the onset of AMI, cTn in the patient’s blood undergoes rapid degradation into smaller molecular fragments. These fragments are predominantly excreted in the urine after crossing the glomerular filtration barrier.^[134]^ Several clinical studies have ascertained the capability of hs-cTns immunoassays to detect cTnI molecules in the urine of patients with AMI.^[135–141]^ Conversely, Mb, owing to its small molecular weight, is promptly released from the injured myocardium and rapidly excreted from the kidneys within 24 hours.^[44]^ Urine serves as an ideal noninvasive sample that can be easily collected in large volumes,^[142]^ offering unprecedented possibilities for the diagnosis of MI. Htun et al^[143]^ demonstrated the potential of urinary peptide biomarkers in predicting future ACS events in asymptomatic patients. Miao et al^[144]^ reported that patients with hypertension and higher urine concentrations of cTnI are more prone to events such as MI than those with normal blood pressure. In a study by Chen et al, 378 patients with diabetes were enrolled, and their fresh urinary hs-TnI levels were measured. The results revealed elevated urinary cTnI levels in patients with type 2 diabetes. According to the results of a multivariate logistic regression analysis, a urinary hs-cTnI level > 4.1 pg/mL was associated with an increased risk of cardiovascular adverse events, such as AMI and HF, during a 3-month follow-up period.^[141]^ Similarly, Streng et al utilized immunoprecipitation and a dual antibody-sandwich ELISA to detect urinary cTnI concentrations in patients with AMI. The study findings demonstrated that urinary cTnI concentrations were significantly higher in patients with ACS than in healthy controls.^[135]^ In a study conducted by Wang et al, urine samples from patients with ACS and healthy controls were subjected to ultra-performance liquid chromatography/mass spectrometry analysis. The findings revealed elevated levels of TnI, CK, and triglyceride isoenzymes in urine samples from the ACS group compared with those in the control group.^[145]^ However, further large-scale studies are required to confirm the role of urinary biomarkers in clinical practice. Specifically, there is currently no published literature on the detection of cardiac-specific cardiac enzyme metabolites (cTnI and cTnT) in urine as diagnostic tools for AMI.

### 4.4. Salivary cTns associated with AMI

Saliva is a colorless, thin, and slightly acidic biological fluid (pH 6–7) that exists in the oral cavity. It is mainly produced and secreted by paired major salivary glands (parotid, sublingual, and submandibular), with a small portion produced by minor salivary glands. Saliva is composed predominantly of water (approximately 94–99%), 0.2% inorganic substances, 0.5% organic substances, and a variety of cellular constituents, including bacteria, enzymes, hormones, antibodies, growth factors, proteins, and genetic molecules. Saliva has many functions, such as wound healing, food softening, swallowing, and maintaining homeostasis in the oral cavity.^[146]^ Compared to the collection of serum samples, saliva collection is simple and noninvasive, allowing for more frequent sample collection. This advantage leads to a reduced risk of clotting, ease of transportation and storage, diminished patient discomfort and anxiety, improved patient compliance, and suitability for individuals with old age, hemophilia, or aversion to venous blood collection.

Additionally, saliva collection significantly reduces the risk of disease transmission, such as human immunodeficiency virus and hepatitis, because it eliminates the potential release of blood pathogens by medical personnel. Thus, salivary testing holds the potential as a viable alternative to blood or tissue testing.^[147,148]^ Saliva, as a “body mirror,” reflects local and systemic health conditions, making it a potential biomarker for detecting oral and systemic diseases.^[149]^ Salivary detection can provide additional diagnostic value for AMI.^[150]^ Mirzaii-Dizgah and Riahi^[151,152]^ identified AMI biomarkers CK-MB, cTnT, and cTnI in the oral fluid of patients using enzyme immunoassays. Furthermore, Miller et al^[153]^ found elevated levels of Mb in the oral fluids of AMI patients. Intriguingly, several previous studies have established a correlation between salivary and blood cTns, CK-MB, and CRP concentrations in patients with MI.^[140,154–156]^ Another study conducted by McDevitt et al^[157]^ showed that salivary TnI concentration in patients with ischemic heart disease was positively correlated with the disease stage. In a previous study involving 47 patients diagnosed with AMI and 15 healthy controls, significantly elevated levels of serum and salivary hs-TnI were observed in patients with MI compared with healthy individuals.^[158]^ Despite the potential of salivary biomarkers such as TnI, CRP, CK-MB, and Mb in diagnosing AMI, their utilization is still in its infancy. Further studies are required to validate these results.

### 4.5. Other potential biomarkers for AMI diagnosis

Irisin, a muscle-secreted protein, is widely present in the myocardium and detectable in saliva. Salivary irisin is synthesized by the plasma and mucus glandular vesicle cells of salivary glands.^[159]^ In an animal model of AMI, Kuloglu et al^[160]^ found a correlation between decreased serum irisin levels and markers such as CK-MB and TnI, indicating reduced irisin production and release in the myocardium, liver, kidney, and skeletal muscle. Similar results were reported by Ozturk et al^[161]^ Aydin et al^[162]^ demonstrated that salivary glands produce and release irisin, and its levels correlate with salivary markers such as TnI, CK-MB, and CK in patients with AMI, suggesting salivary irisin testing as a potentially superior diagnostic indicator compared to serum irisin. In addition, several plasma biomarkers, such as interleukin 6, hs-CRP, B-type natriuretic peptide, copeptin, myeloperoxidase, transforming growth factor-15, pregnancy-associated plasma protein, soluble CD40 ligand, and tumor necrosis factor, have shown elevated levels in the plasma of AMI patients. These factors may serve as potential biomarkers for the diagnosis and prognostic prediction of MI in the future.^[163–166]^

## 5. Summary and prospect

AMI remains a significant global health challenge. ECG is widely used as the most common/initial diagnostic tool, realize ECG as soon as possible, preferably in the prehospital setting, which allows in case of specific findings to start thrombolysis according to the time of diagnosis and delay to go to cardiac facility for coronarography and angioplasty, and cardiac-specific biomarker testing is generally used in patients with unremarkable ECG changes and atypical symptoms. Currently, traditional biomarkers have shortcomings, so the search for novel biomarkers is crucial for the early diagnosis of AMI. Recent progress in medicine and biotechnology has led to the discovery of numerous biomarkers for MI that exhibit improved tissue specificity and sensitivity. These advancements have facilitated more precise clinical diagnosis, effective treatment, and enhanced prognosis of AMI. However, no single marker currently fulfills the characteristics of an ideal biomarker. This article now provides a detailed review of miRNAs, H-FABP, urinary cTns, salivary cTns, and other biomarkers used in the early diagnosis of AMI, demonstrating that new biomarkers could help reduce the delay in diagnosis and facilitate the recommended treatment. For the novel biomarkers, relatively few clinical trials have been conducted and these have not yet been translated into actual clinical practice. Therefore, more detailed, large-sample and statistically based studies are needed to establish their reliability, sensitivity and specificity. This will enable them to play a crucial role in clinical practice and provide prompt and effective treatment for patients with CVDs, thereby reducing the mortality associated with AMI.

## Acknowledgments

We thank Bullet Edits Limited for the linguistic editing and proofreading of the manuscript.

## Author contributions

**Writing – original draft:** Xuelan Huang.

**Writing – review & editing:** Yumei Luo, Suwen Bai.
